# Note on the Triplex Pill of Dr. John W. Francis

**Published:** 1873-11

**Authors:** Edward R. Squibb


### NOTE ON THE TRIPLEX PILL OF DR. JOHN W. FRANCIS.

BY EDWARD R. SQUIBB, M. D.

Many years ago the well-known Dr. John W. Francis used a pill which he called triplex, and which became so popular as to be widely known beyond Dr. Francis's own locality. It originally consisted of equal parts of socotrine aloes, scammony, and mercurial pill mass, in a pill of three grains.

During the latter part of the professional career of Dr. Francis, he modified the formula for the then well-known triplex pill, and the modification was generally accepted as an improvement. The pill was then long prescribed by physicians and used by hereditary transmission to families simply as triplex pill, and its composition neglected and forgotten except by the pharmacists who made them, and finallly the pharmacist who principally served Dr. Francis before his death, alone had the formula.

Some ten years ago, when the writer was serving as a committeeman of the New York Academy of Medicine in preparing a report on the United States Pharmacopœia, previous to its revision, he applied to the son of Dr. J. W. Francis, the present Dr. Samuel W. Francis, for the improved formula of his father, with a view to its republication. Dr. S. W. Francis examined his father's papers, but without finding the formula, but informed the writer that Messrs. Delluc & Co., pharmacists, had made the pill for his father, and continued still to make and sell them. The writer then applied to Mr. Delluc for the formula, but it was refused, and the subject was dropped.

Recently, however, Dr. Samuel W. Francis, in a note to the writer, sent the formula for the pill, saying that he had obtained it from Messrs. Delluc & Co., and communicated it for publication.

Although the pill, under the close policy of Messrs. Delluc & Co., has declined in its general use of late years, it is still highly valued by many physicians as one of those happy combinations which grow out of the long experience and fertile resources of eminent men in the professon of past years. When it is remembered how successful those men were in their practice, and hence what a hold upon the confidence of the community they have handed down to the profession of the present day, it does not seem wise to lose or disregard the agencies by which they reached their benificent results.

The formula of Dr. Francis's triplex pill is as follows;

Take of Powdered Socotrine Aloes, Powdered Scammony, Mercurial Pill Mass, each 1 troy ounce; Croton Oil, 20 minims; Oil of Carraway, 1½ fluid drachms; Tincture of Aloes and Myrrh, or Elixir Proprietatis of Paracelsus, 2 fluid drachms.

Mix the ingredients well together by very thorough trituration, make a pill mass, and divide the mass into four hundred pills.

The usual aperient or laxative dose is one pill at bedtime until the natural condition is restored.—*Proceedings Am. Phar. Association*, 1872.

